# The Role of Religious Culture in Medical Professionalism in a Muslim Arab Society

**DOI:** 10.5334/pme.920

**Published:** 2023-02-24

**Authors:** Haythum O. Tayeb, Ara Tekian, Mukhtiar Baig, Harold G. Koenig, Lorelei Lingard

**Affiliations:** 1 Faculty of Medicine, King Abdulaziz University, Jeddah, Saudi Arabia; 2 Department of Medical Education, University of Illinois, Chicago, USA; 3 Faculty of Medicine in Rabigh, King Abdulaziz University, Rabigh, Saudi Arabia; 4 Departments of Psychiatry and Medicine, Duke University Medical Center, North Carolina, USA; 5 University of Western Ontario, Ontario, Canada

## Abstract

**Purpose:**

Calls have been made to integrate concepts and practices derived from Muslim culture into medical professionalism in Muslim societies. Little is known about how these religious cultural concepts (RCCs) influence medical practice and education. This study explored the influence of RCCs on medical professionalism in Saudi Arabia.

**Methods:**

This was a qualitative study that implemented a constructivist, grounded theory approach. Semi-structured interviews about RCCs and medical professionalism were conducted with 15 Saudi physicians at a single academic medical center. Purposive sampling was used to recruit participants of different genders, generations, and specialties. Data collection and analysis were iterative. A theoretical framework was formulated.

**Results:**

Key findings: (i) the role of RCCs in medical professionalism is perceived to be constantly evolving due to the evolution of societal interpretations of RCCs; (ii) participants described applying two standards to judge what is professional: a medical standard and a religious cultural standard. Participants shifted between these two standards variably and non-linearly. This variable shifting altered the values shaping medical professionalism, at times unpredictably.

**Discussion:**

Academic Saudi physicians argued against assuming a stable traditional interpretation of RCCs, emphasized the evolving contribution of RCCs to medical professionalism, and indicated that the process of merging religious cultural and medical standards in medical practice is variable and may alter medical practice values. Therefore, these physicians perceived RCCs to be useful as supplements to but not as a backbone for medical professionalism. Careful consideration of the potential impact of RCCs on the values of medical professionalism is warranted.

## Introduction

Research on the topic of religion and health has accumulated to a point where clinicians can no longer ignore this area of a patient’s life [1]. Findings from numerous quantitative studies indicate a need to train the next generation of physicians to pay more attention to the role of religion in medical practice. Nevertheless, how to integrate a patient’s religious faith into their care, and the extent to which such beliefs should influence medical decision-making, has not yet been fully worked out [2]. Even in a highly religious society such as Saudi Arabia, there are disagreements among health professionals on how to best integrate a person’s religious beliefs into their care, while simultaneously maintaining the boundaries and rules of medical professionalism [3].

For example, a spirited debate on medical professionalism took place among a group of Saudi physicians in a health professions education class. After a thorough discussion of approaches to define medical professionalism, a learner suggested that the best way to define medical professionalism in Saudi Arabia was to rely on a Muslim religious perspective. The argument was that the Holy Quran and Prophet Muhammad’s narrations and biography provided an excellent portrayal of attributes that are the essence of professionalism. A counterargument emerged, positing that defining medical professionalism from a religious perspective may be fraught with problems. The professor facilitating the class terminated the heated discussion, suggesting a 10-minute break, deliberately leaving a relevant but sensitive debate unresolved.

How might medical professionalism be optimally defined? This question has been asked since early times when physicians assumed predominantly spiritual healer roles [4]. Today, physician roles and attributes are described in standard frameworks such as the American Board of Internal Medicine (IM) Physician Charter and the Canadian Medical Education Directives for Specialists (CanMEDS) [5,6]. Recently, there have been concerns that the standardized medical professionalism frameworks based predominantly on Western values may not fit well in non-Western cultures [7,8,9]. Calls have been made to revise medical professionalism frameworks in these societies so that medical professionalism more aptly reflects their unique values and contexts [10,11,12,13].

In Muslim Arab societies, Islamic teachings are pervasive, and concepts and practices derived from Muslim culture are interwoven into the fabric of national cultures. There have been efforts therefore to integrate these religious cultural concepts (RCCs) into medical professionalism. Al-Eraky et al. described four “gates” to medical professionalism, including “dealing with God” as the overarching concept. They defined some medical professionalism attributes using religious terms such as *taqwa* (avoidance of bad deeds for fear of God) and *ehtisab* (performance of good deeds for the sake of God) [14]. Abdel-Razig et al. developed a consensus statement that defined medical professionalism in the United Arab Emirates context [15]. The statement described medical professionalism from a moral religious perspective using terms such as *itqan* (pursuit of perfection inspired by higher motives) and *ihsan* (manifesting faith-driven values in behavior) while emphasizing value differences between Muslim Arab and Western societies. The Saudi Medical Education Directives Framework (SaudiMED) [16], a competency-based framework that serves as a standard for evaluating Saudi medical graduates, includes belief in “prophetic medicine” and the application of Islamic ethics as two of its unique sub-competencies [17].

Although it is important that medical professionalism frameworks in Muslim Arab societies integrate RCCs into medical professionalism to reflect the socioreligious milieu that hosts all social transactions in these societies, it is unclear how this integration should be pursued and how it might influence medical professionalism. What will medicine look like if medical professionalism is defined from a religious perspective? What roles do RCCs play in medical practice in Muslim Arab societies? Will such medical professionalism frameworks maintain the standard core values of medicine described in professionalism charters? Saudi Arabia is an example of a Muslim Arab society where RCCs play a strong role in social transactions, including an influence on medical professionalism [16,17].

The present study sought to provide answers to the above questions from the perspectives of Saudi academic physicians practicing in the religious cultural milieu of Saudi Arabia. Saudi Arabia is a socially conservative country that is home to Islam’s holiest sites. It is also a country that has seen a dynamic interaction between its religious tradition and modernity, enabled by socio-political changes that have taken place over the past several decades. In Saudi Arabia, most social transactions, including those in medicine, may be readily influenced by this interaction of religious cultural and other modern concepts. The aim was to formulate a theoretical framework for the role of RCCs in medical professionalism in a Muslim Arab society. This study sought to provide a way forward in discussions of this type for medical educator, that will become increasingly important in the days ahead both within the Middle East and other areas of the world, including the West.

## Materials and Methods

This study was approved by the Ethics Review Committee at the Faculty of Medicine at King Abdulaziz University. An interpretivist constructivist grounded theory (CGT) approach was used to explore the social process of how RCCs may shape medical professionalism in a Muslim Arab society. The purpose was to generate a narrative account of this process and develop a theoretical framework grounded in participant reality while accepting the role of researchers in co-interpreting and co-constructing the data [18]. Participant recruitment took place from 2017 to 2020 from a single academic center in Jeddah, Saudi Arabia (King Abdulaziz University Hospital). We recruited Saudi academic physicians who were well-informed about both standard medical professionalism conceptualizations and the religious cultural influences in Saudi Arabia. We therefore chose physicians who are Saudi, Muslim, and completed postgraduate training abroad in North America, Australia or Europe, where they would have been exposed to a practice setting where standard Western conceptualizations of medical professionalism are applied without an explicit Islamic religious cultural influence. This sample was purposefully chosen to elicit comparative insights with regards to how medicine is practiced in both settings. Inclusion criteria were: 1) agreed to participate in the study; 2) successful completion of a residency or fellowship; 3) completion of at least one year of medical training at an international institution in North America, Europe or Australia; 4) at least two years of clinical practice in Saudi Arabia; 5) familiarity with Saudi religious culture, evidenced by having lived for an extended period in the country; and 6) having leadership roles in undergraduate or postgraduate programs that exposed them to issues related to medical professionalism in practice and education. Sampling of leaders in medical education was intended to elicit the insights they gleaned from dealing with issues related to medical professionalism in the contexts of undergraduate education and postgraduate training. Using purposive sampling, we recruited participants from both genders to elicit views related to gender relations and women’s rights; from both junior and senior faculty members to explore generational differences; and from medical and surgical backgrounds to explore the role of RCCs in these two practice contexts. Aiming to explore potential conflicts between RCCs and medical standards, we also recruited participants with different degrees of Islamic conservatism based on the first author’s subjective impression from prior personal encounters with participants, which is consistent with the study’s constructivist approach.

One-on-one, semi-structured, 60-minute interviews with participants were conducted either in-person or via remote video teleconferencing. The interview guide is presented in Online Appendix 1. The interviews began with a brief introduction explaining that the overall goal of the study was to explore the nature of medical professionalism in the Muslim Arab society of Saudi Arabia and to understand how this related to medical education. We then asked open-ended questions to elicit participant views on how medical professionalism should be defined in Saudi Arabia with reference to both practice and medical education. Probing questions focused on aspects related to the role of RCCs in medical professionalism, medical education, and how participants integrated RCCs into their practices. The interviews took place mostly in English as these participants spoke English fluently. Participants occasionally shifted to Arabic whenever they felt they needed to. The interviews were transcribed using pseudonyms to maintain participant anonymity. The occasional Arabic parts of the interviews were transcribed verbatim in Arabic and translated to English. All interviews were conducted by the first author except for one interview that was conducted by the last author. The first author is an alumnus of and an associate at the same institution participants were recruited from. The interviews proceeded as a conversation between two faculty members interested in medical professionalism, without a power relationship between the conversing parties. Data analysis followed CGT procedures, i.e., was conducted in an iterative fashion as data were collected, refining interview probes as themes were identified. Open, axial, selective and theoretical coding were used to identify concepts and categories. Analytic memos were documented. A code book that defined categories and identified their boundaries was developed. Through regular meetings and correspondence, authors shared in data analysis, reviewing the categories and concepts as they evolved, exploring reflexivity, and discussing the developing theoretical framework. Recruitment stopped after the 15^th^ interview as themes were sufficiently defined and illustrated to support a cohesive theoretical framework. After a framework was formulated, 30-minute member checking meetings were held with four participants [18,19]. During these meetings, the study results were summarized and framework described, taking into account participant feedback, resulting in minor refinements.

## Results

### Overview

A total of 15 participants (10 males) were recruited. The proportion of females was lower than males because fewer females fit the inclusion criterion of leadership roles. Participant ages ranged between 35 and 60 years. Nine participants were from medical and six were from surgical backgrounds. Five were assistant professors, seven were associate professors, and three were full professors.

The data revealed how recent sociocultural changes in Saudi Arabia have led to an evolution and modernization of how traditional RCCs have been interpreted and thus integrated in medical practice. Participants thought RCCs remained relevant to medical professionalism and could be useful as a supplement to a backbone of generically described medical professionalism. When integrating RCCs into medical practice, participants reported applying two standards to judge what is professional— a medical standard and a religious cultural standard. They described shifting variably and non-uniformly between the two standards in a non-linear fashion, at times unpredictably. The use of this dual standard sometimes altered the values that shaped their medical practice.

**Figure 1:**
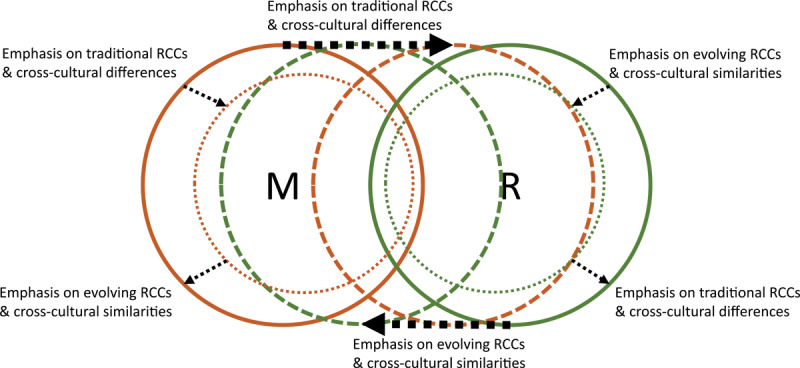
A Venn diagram describing the overlapping and dynamical relationship between the religious cultural standard and the medical standard. The circles represent the medical standard (M) and the religious cultural standard (R). The two standards overlap in what they dictate but not completely. The two standards may move toward or away from each other so that larger or smaller areas of what they dictate overlap. Respect for religious tradition and emphasis on cross-cultural differences reduce the area of M that is not congruent with R whereas respect of the evolving nature of religious culture and emphasis of cross-cultural similarities reduce the area of R that is not congruent with M. The areas of M and R may increase to subsume the other standard. Emphasis on tradition and differences increases the area dictated by R whereas emphasis on evolution and values increases the area dictated by M, and vice versa.

**Figure 2:**
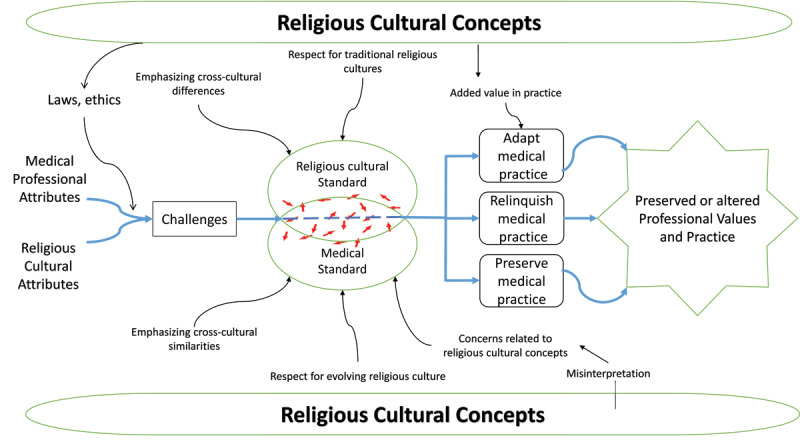
A theoretical framework generated from the data, describing the dynamic of merging of medical professional attributes with religious culture ones, and shifting between standards of MP to shape medical practice values. RCCs shape the practice milieu of Saudi Arabia, contributing to laws and ethics and adding value. They also create cultural differences. Emphasis on cross-cultural differences and religious traditions facilitate applying the religious cultural standard whereas emphasis on cross-cultural similarities and the religious facilitate adherence to the medical standard. Misinterpretations of religious concepts were perceived to be a source of concern.

The coding matrix summarizing the theoretical codes is provided in Table 1 along with illustrative quotes. The theoretical framework generated from the data is presented in Figures 1 and 2.

**Table 1:** Coding matrix showing the organization of theoretical codes and concepts.


THEORETICAL CODING		#	ILLUSTRATIVE PARTICIPANT QUOTES

Belief in medical professional attributes		1	You can think of medical professionalism as basic human behaviors like dignity, honesty, truthfulness, discipline … Compassion, being a good listener, being sincere, and being able to build rapport. – P7

Respect for evolving religious culture	Tradition	2	There are cultural values that we have to respect … but the religious point of view is [important] for Muslim … Such values are red lines that we shouldn’t cross – P14

		3	National professionalism depends on culture. Eye to eye contact, in the west, indicates respect between a man and a woman. In our civilization it may mean something we should avoid. – P5

	Evolution	4	People started to open up in the past years … Things that used to be restricted in the name of religion are now ok! Religious interpretation … evolves. – P8

		5	In the past there was the issue of women covering their faces and hijab and whether the doctor is allowed to see them or not. Now, the patient comes in and they know it is OK. – P10

		6	Patients now come asking for the female physicians! – P13

		7	“Currently … there are the people that will say: man, don’t bring religion into [a medical discussion] … Show me with studies and convince my brain. – P6

Value of RCCs in medicine	Added values	8	It will be much easier for physicians to understand and respect the medical concept when you connect it to Islamic teaching… they will be more likely to comply and respect it. – P1

		9	Do I sometimes use religious terms when I talk to patients? I think I do and I think it’s reasonable … Because … they find it helpful to hear:… God willing this medication would help you. – P12

	Concerns	10	I don’t think it’s helpful to label things [with religious terms] because people [get] polarized. – P9

		11	How are you going to measure *taqwa* (doing good for Allah’s sake)? You cannot! – P3

		12	The next generation will be even faster … communicating with the world … If we do not go to the right concepts, we will be outdated. – P15

		13	If I don’t follow [medical professionalism defined by RCCs] … am I going to be considered a *kafir* [disbeliever]? – P7

Conflicts and challenges		14	Someone would pray that [I’m cursed] because I told their dad about the [troubling] diagnosis … He would yell: “have *taqwa!* Be afraid of Allah! You are killing my father!” – P8

		15	A patient has a mass and you tell them they need a biopsy and they say I prayed istikhara [a prayer to seek God’s guidance] and I’m happy with not doing the biopsy. If you disagree with that, they would say but God will take care of me. – P12

		16	I say we don’t have enough evidence … to support *hijama* [religiously inspired cupping] … But [patients] look at me with wide eyes and say: doctor, Prophet Muhammad said to do this! – P13

		17	Even clinicians disagree with brain death … how can you ethically deal with that when you have someone brain dead and the family wouldn’t allow you to pull the plug? – P4

		18	For non-Muslims, I will [offer this treatment]. For Muslim patients I rely on an Islamic *fatwa* (ruling) that does not allow this treatment… – P14

Shaping practice	Adapting medical practice	19	A patient was telling me she wanted to try *hijama* instead of [medical treatment]. There was no harm to delay the treatment a bit in this case. – P14

	Preserving medical practice	20	The mother wanted to [delay treatment] … and a [religious healer] to treat her daughter. I respected their expectation … but it was my duty to correct them because it interfered with patient care. – P1

	Relinquishing medical practice	21	Islamic scholars define [suitability of a treatment]. Beyond that … I cannot do it because of my professional belief. You could find another physician or go to another country” – P15

		22	Life is in Allah’s hands and it’s mine to take [through euthanasia or abortion]. – P2

Finding the gold standard	Religious cultural gold standard	23	We are restricted by Islamic juristic laws … religious ruling is number one for me. – P14

	Medical gold standard	24	If all scientists or physicians agree [an intervention] will help humanity, I will keep digging in my religion to find a solution or explanation from the religion’s point of view. – P13

	Shifting & merging	25	If you found a conflict between the two [religion and medicine], what it’ll end up being is that one of the two got misinterpreted … So, you reconcile the two so that you don’t show conflict. – P6


#### Defining Professionalism in an Evolving Religious Culture

To define medical professionalism, participants used standard professional attributes (Table 1, quote 1) but endorsed integrating traditional RCCs into medical professionalism (Table 1, quotes 2, 3, 8, 9). One participant described the need to *“think global and act local. We should have guidelines … but then we also have our local societal, religious factors”* (P4). *“Anchor[ing]” (P1)* medical professionalism to RCCs was thought to bolster adherence to medical professionalism. Examples of RCCs endorsed as relevant to medical professionalism included “*awrah”* (P4) (that which must be covered from others), *ihsan* (P1) (seeking perfection for the sake of God), *“dara’ almafasid”* (P15) (preventing harms), and *“Allah’s fate and Allah’s rewards”* (P13) as consoling concepts for patients dealing with bad news.

In addition to endorsing traditional RCCs, however, participants emphasized that RCCs have noticeably evolved during recent decades, thereby altering social transactions within the practice of medicine (Table 1, quote 4). One example has been the easing of religious rules shaping gender boundaries, which has made it more acceptable to have females examined by male physicians (Table 1, quote 5) and to have females work as healthcare professionals (Table 1, quote 6). Another perceived change has been the language patients expect during explanation of medical issues. Participants described reduced emphasis on RCCs and increased emphasis on medical and scientific explanations of medical information (Table 1, quote 7).

### Positioning RCCs in Medical Professionalism Frameworks

Although participants emphasized incorporating RCCs into medical professionalism, they argued against using RCCs as the predominant language defining medical professionalism (Table 1, quotes 10 and 11). A medical professionalism framework relying entirely on RCCs was felt to potentially be unappealing and outdated (Table 1, quote 12). In addition, *“religious terms are subject to a lot of interpretations”* (P8) by different religious schools and by non-Arab and non-Muslim practitioners, which may hinder their applicability and universal acceptance. Some participants even worried that using RCCs to judge professionalism may lead to judgments about the practitioners’ religiosity (Table 1, quote 13).

Instead, participants thought RCCs may be added to medical professionalism standards as *“supplemental elements”* (P6) that provide context *“in the introduction or background”* (P5). This would allow harnessing their values, mitigating concerns, and not *“reinvent[ing]the wheel”* (P7).

### The Dual Standard

Despite believing that RCCs and medical professionalism were largely consistent, participants reported challenges that arose when attempting to integrate RCCs into medical professionalism. In some instances, a religiously inspired behavior may conflict with standard practice (Table 1, quote 14). RCCs sometimes influenced medical decisions, leading to unproven or delayed treatments, ethical dilemmas, or disparities in treatment access (Table 1, quotes 15, 16, 17, 18). Some medical practice regulations were felt to be influenced by RCCs, potentially restricting access to otherwise acceptable treatments (Table 1, quote 18). In these challenging situations, participants often succeeded in adjusting their practice to accommodate RCCs without infringing on professional values (Table 1 quote 19). In other instances, physicians refused to integrate RCCs and instead sought to preserve standard medical practices to prevent patient harm (Table 1, quote 20). In yet other instances, physicians relinquished the standard practice and submitted to religious cultural demands despite compromising medical practice values (Table 1, quotes 21, 22).

To choose a course of action in such situations, participants indicated that they would apply two standards, a medical standard and a religious cultural standard (Table 1, quote 23, 24). The premises of the two standards were often overlapping. When they were not, however, participants had to reconcile them (Table 1, quotes 25, 26). Participants viewed the religious cultural standard as inclusive of the basic premises of the medical standard, expressing that Islam *“should mould over anything good”* (P6). If a medical practice was incompatible with an RCC, participating physicians indicated they would *“keep digging in religion to find a solution from the religion’s point of view”* (P13). Such resolution was difficult to achieve, however, in situations where a medical practice was not consistent with a religious ruling (Table 1, quote 18, 21). When a religious ruling might need to be reconsidered for medical reasons, they would “*discuss cases … with Islamic scholars”* (P15), seeking approval to follow the medical standard.

As such, physicians shifted back-and-forth between the religious and medical standards. This shifting was a variable, non-uniform, and non-linear process, which introduced a variability in how medical professionalism shaped practice, at times unpredictably. This was illustrated by a participant’s account of a family compelling him to hide a troubling diagnosis from their loved one: *“They made me swear to God so I could not tell her!”* (P10). The participant later regretted hiding the diagnosis but had to abide by the oath he swore. In an attempt to circumvent the oath, he said *“I beg you to see another doctor!”* (P10). Subsequently, he eventually decided to disclose the diagnosis and did so skillfully. Another example of this dynamic was when a participant described how a mother considering a legal abortion might be counselled. *“You could go either way”* (P15), the participant said, referring to how one could offer this medical service, or alternatively, might try to convince the patient that *“this [pregnancy] may be a door for getting rewards from Allah by being patient”* (P15). These stories illustrate how this non-linear shifting between the religious cultural and medical standard sometimes affected how physicians counselled patients and made clinical decisions.

### Theoretical Framework

A theoretical framework describing the role that RCCs play in medical professionalism in the Muslim Arab context was developed based on the findings from this study (Figures 1 and 2). The framework describes how medical professionalism and religious culture merge within a practice environment shaped by RCCs. This merging is sometimes challenging, leading physicians to engage in the process of shifting non-linearly between the two standards they must abide by.

## Discussion

This study reveals how the role of RCCs in shaping medical professionalism in Saudi Arabia has evolved over time, which reflects the cultural modernization of Saudi society [20]. The study illustrates the process by which Saudi academic physicians incorporated these RCCs into their practices. In most situations, they relied heavily on personal experience, training and understanding of religious culture to successfully meet the demands of both RCCs and medical professionalism. In situations where these demands were divergent and competitive, physicians would resort to discussions with peers, ethics committees, and religious scholars to reach a resolution. That process, though, can be relatively haphazard. This is because these physicians would non-uniformly, non-linearly and variably shift back and forth between the obligations of medical and religious cultural standards, a variability that has been previously described when physicians deal with issues related to religious or politically charged matters related to medicine [21]. This introduces a degree of unpredictability, potentially allowing medical practice and its values to change unintendedly. This may lead physicians, for example, to restrict certain treatments or even risk discriminating on religious grounds. At stake is nothing less than the physicians’ ability to remain patient-centered and adherent to professional standards.

This concerning finding should be viewed in light of present-day controversies surrounding the integration of religion and medicine. Separating religion and medicine may be difficult given their overlapping roles as responses to human suffering [22,23]. Even in Western cultures, physicians opinions and stances on issues related to religion may influence their practice and their approaches to medical education [21]. It has been argued, however, that religion’s non-empirical basis may make it incompatible with secular medicine [24,25]. While religion serves an important role in the ethical valuing of medical practices, it has been shown that religious moral grounds may be used to restrict access to legally available treatments [26]. Outright rejection of RCCs, in turn, may show a lack of respect for societal and patient preferences to integrate RCCs into medical professionalism [27]. However, one cannot assume that integrating religion and medicine is preferrable for most members of society [28,29], or that RCCs provide input that is inherently superior to that of other sources shaping medical professionalism [30]. Physicians should respect and, where possible, accommodate individual and societal religious views. However, there is some risk of introducing inauthenticity in clinical interactions if physicians adopt religious views they do not believe in, and there may even be a risk that religious concepts are used to coerce patients into medical decisions those patients would not accept otherwise [29].

Participant accounts echoed some of these concerns, and we argue they should be considered when evaluating proposals for modifying medical professionalism paradigms in Muslim countries. Proposals by Eraki [14] and Abdel-Razig [15] and colleagues place RCCs at the center of medical professionalism definitions and use religious terms to describe professional values and attributes. In contrast, this study found that placing RCCs at the center of medical professionalism would base medical professionalism on concepts that are too hard to define, too prone to being misinterpreted, too likely to be variably applied in practice, and too difficult to measure. The contrast between this study and prior studies may be attributable to differences in methodology and sampling. This study focused on perceptions of Saudi physicians who were trained abroad and have comparative insights between practice in Muslim Arab and Western contexts, which may have made the sample more sensitive to potential problems that may arise from placing RCCs at the center of medical professionalism. However, Saudi Arabia is perhaps one of the most conservative Muslim countries despite recent socio-political changes. If a medical professionalism based on RCCs were to be appropriate anywhere in the Muslim Arab world it would be in the Saudi context. In spite of that, our participants maintained the contrary. We contend, therefore, that further scrutiny is required before adopting a medical professionalism definition that is based on RCCs, and that the optimal position of RCCs in medical professionalism paradigms is to include them as supplements that would enhance physicians’ understanding and adoption of widely accepted universal medical professionalism standards. There is research from the region whose findings are consistent with this suggestion, showing general acceptance of widely acknowledged standards for medical professionalism, such as the Arabian LAMPS professionalism inventory and the SaudiMED framework [17,31,32,33,34]. This debate on where to place RCCs in medical professionalism paradigms must be resolved before more aggressive integration of RCCs is pursued.

### Implications for Medical Education

RCCs already serve important roles in defining medical professionalism in Saudi Arabia. Therefore, educators should consider how best to address the relationship between RCCs and medical professionalism in medical education in this country and other countries in the Middle East where religion is such an important social force. Not doing so risks leaving RCCs to serve as a hidden curriculum taught implicitly and unintendedly [31,35]. This challenge of integrating religion and spirituality into the medical curricula has been a global one [36,37]. Although educators and medical associations have advocated for integrating spirituality into undergraduate and postgraduate medical education, there is wide variability in the extent to which this has been embraced [38,39]. In Saudi Arabia, all medical schools teach medical professionalism and ethics in their curricula, but these curricula are variable, and it is unclear how much is taught about RCCs relevant to medical professionalism [40].

Therefore, introducing RCCs into professionalism curricula remains a challenge. Some progress has been made, however. Experts have advocated that “spiritual competencies” be established, i.e., a set of spiritual (including religious) learning objectives applicable to medical practice [37,41]. At the level of cognitive and affective learning objectives, RCCs have been introduced into medical professionalism curricula at different stages of undergraduate and graduate training as part of the “cognitive base” for medical professionalism, which is typically taught in the early years of medical school [11,36,42]. Findings of the current study support the inclusion of cognitive and affective objectives with the aim of teaching physicians the importance of respecting Islamic RCCs as part of the larger goal of cultural competence in medical practice [43]. In the Saudi context, an effort has been made to summarize aspects of medical ethics related to Islamic jurisprudence [44]. Nevertheless, simply “hearing about” the importance of integrating spirituality/religion into patient care is insufficient [37]. Rather, it is argued that skills on how to do so be introduced into medical curricula. The aim would be to train physicians to deal with religious and spiritual concepts that arise in clinical practice. Nevertheless, how much physicians should engage in such endeavours remains controversial [45]. Skills in this category include spiritual history taking and spiritual support, and instructional methods include shadowing role practitioners, seminars, and workshops that include role-playing [41,46]. Educators have proposed that such skills may be introduced as competencies or entrustable professional activities (EPAs) [4,13]. The present study found that RCCs are perceived to be subjective, evolving, non-universal, and laden with potential concerns when overly emphasized in medical practice. This may limit translating them effectively into educational materials and frameworks. Therefore, rather than specifying RCCs as specific competencies or EPA, we recommend introducing RCCs as abstract concepts at macro levels in medical professionalism curricula, linking them with concepts of cultural competence and patient-centered care [43].

The question of how to assess elements of medical professionalism that flow from non-Western cultures remains insufficiently addressed by empirical studies despite the increase in the number of publications related to the influence of some of these cultures on medical professionalism [47]. If RCCs are introduced to medical curricula as supplemental concepts that inform relevant aspects of medical professionalism and cultural competence, then they should not be assessed in isolation. Their assessment should rather be linked with the blueprints emerging from principles of standard medical professionalism since these represent the overarching goal of including RCCs in medical curricula to begin with.

### Quality, Credibility and Limitations

Following a constructivist, postmodern approach to qualitative data analysis, this project’s results and conclusions are anchored in Charmaz’s criteria of credibility, originality, resonance, and usefulness [18]. Evidence of the credibility and originality is derived from the data’s richness, depth, and range. The data emerging from this study reveal in-depth accounts of complex sensitive topics related to professionalism and religion, including the discussion of religiosity, modernity, and controversial medical and alternative treatments. The theoretical framework developed here links medical professionalism with RCCs in an explicit and complex manner that may raise challenging questions about the preservation of medical practice values. The credibility of this theory and framework is also derived from the methods used to generate them, including keeping an audit trail, writing frequent analytic memos, seeking disconfirming evidence, and theoretical sampling to verify and aid in theory development. The study’s findings resonated with its participants during member checking and are expected to be useful for experts considering medical professionalism frameworks and curricula.

However, this study also has limitations. We recruited Saudi academic physicians from a single institution in one city in the Western province of Saudi Arabia. CGT findings are never generalizable – in our case, neither to the rest of Saudi Arabia nor to the broader Muslim Arab world. The findings may not reflect those of non-Saudis practicing in Saudi Arabia. In addition, the framing of this study was to compare the experiences of Western-trained Saudi physicians in the Saudi setting with their experiences in Western practice settings. Alternative framings where focus would have been on comparing to other cultures may have led to different responses. Therefore, scholars seeking to ascertain the transferability of our findings will need to consider how our study’s context and sampling relates to their situation. The constructivist approach, in the context of a sensitive issue such as religion, inevitably shapes the findings, as the interviews will have been coloured by how both individuals perceive one another’s medical professionalism and RCC beliefs. The interview script used open ended questions to encourage a wide range of responses, and detailed probing to explore the logic behind the answers, whether or not they were congruent with the interviewer’s views. The relatively lower proportion of females participating in the study was not felt to be a major limitation with respect to the integrity of the concepts and frameworks that emerged, but further exploration of gender-sensitive issues in the future may be of interest for future work. Finally, this study addresses the overall role of RCCs in medical practice without focusing on individual RCCs, a question that should also be addressed in future work.

## Conclusions

The present study revealed how academic physicians practicing in Saudi Arabia may find themselves engaging in a complex process that involves negotiating between RCCs and the standards of medical professionalism. This does not only involve complying with religious cultural traditions, but rather to keeping up with the changing Saudi religious culture. The identification of changes in how Saudi academic physicians interpret RCCs and integrate them into medical professionalism is a key contribution of this study. The findings suggest that we cannot assume a stable, traditional impact of RCCs on medical practice and values, resonating with literature documenting the impact of religious thought on the ethical values of medical practices as it has evolved throughout history [48], including experiences in the Middle East [49,50]. This study also demonstrated that Saudi physicians’ merging of RCCs and medical professionalism is sometimes a haphazard process that may unpredictably alter the values that shape medical practice. This argues against positioning RCCs as the backbone of medical professionalism and underscores the importance of carefully considering the interaction of RCCs and medical professionalism when RCCs are introduced into medical professionalism frameworks and curricula in Muslim Arab societies.

## Additional File

The additional file for this article can be found as follows:

10.5334/pme.920.s1 Online Appendix 1. Final version of the interview guide used in this study.
