# Supplementary material for: The Role of Religious Culture in Medical Professionalism in a Muslim Arab Society
Source: Perspect Med Educ. 2023 Feb 24;12(1):56–67. doi: 10.5334/pme.920 (PMC9997109; doi:10.5334/pme.920)
Supplement: Online Appendix 1. — Final version of the interview guide used in this study. [file pme-12-1-920-s1.pdf]

Appendix

Final version of the interview guide used in this study

Opening questions
1. How would you describe ideal MP? Where do you draw your definition of professionalism from?<br>2. How is MP different for physicians practicing in a Muslim Arab society compared to a Western society?<br>3. How do religious concepts interact with MP in a Muslim Arab society?<br>4. How may RCCs be used in the definition of MP in Muslim Arab societies?
Probing questions
1. How may RCCs interact or augment MP in Muslim Arab societies?<br>2. How may RCCs conflict with MP, if at all? What are pros and cons of integrating RCCs into MP, if any?<br>3. How have the interpretations of RCCs in the context of MP changed over the years? How do different generations interpret them differently?<br>4. In what ways may the following issues be influenced by RCCs: the doctor-patient relationship, gender roles in medicine, confidentiality and patient autonomy, end-of-life decisions, religious-inspired practices?<br>5. What are differences in MP between Muslim Arab societies and Western societies? What roles do RCCs play in either of these settings?<br>6. What are your concerns, if any, in relation to the role of RCCs in MP? How do you assess potential for discrimination, unequal access to care, infringing on patient autonomy, or biases in judging medical decisions?<br>7. How do we reconcile differences between how different groups interpret RCCs (e.g. sunni vs shiite; more observant vs less observant…)?<br>8. How might non-Muslim physicians understand a definition of MP that RCCs? How may the practice of Muslim physicians dealing with non-Muslim patients be influenced by RCCs?<br>9. How may RRCs be integrated in MP frameworks as some literature suggested? What do RCCs like Taqwa and Ihtisab mean to you in relation to MP?<br>10. How may RCCs related to MP be taught and assessed in medical school and training programs?
